# Engineering‐Modulated Molybdenum Enzymes Strategy for Tumor‐Specific Metabolic‐Immunotherapy

**DOI:** 10.1002/advs.76726

**Published:** 2026-07-23

**Authors:** Xiaoxiao Pan, Zifan Pei, Jie Wu, Nan Jiang, Qian Li, Yuqi Yang, Jie Cao, Yechen Huang, Shumin Sun, Qialu Du, Zhicheng Liu, Lin Zhang, Fei Gong, Jinhua Zhou, Liang Cheng

**Affiliations:** ^1^ Institute of Functional Nano & Soft Materials (FUNSOM) Biomedical‐BasicResearch‐Center (BBRC) of Jiangsu Province Soochow University Suzhou China; ^2^ Department of Interventional Radiology The First Affiliated Hospital of Soochow University Suzhou China; ^3^ Department of Obstetrics and Gynecology The First Affiliated Hospital of Soochow University Suzhou China

**Keywords:** cGAS‐STING, metabolic‐immune therapy, molybdenum enzymes, T‐cell activation, uric acid

## Abstract

Molybdenum enzymes and one of their catalytic products, uric acid (UA), play important roles in T‐cell activation; thereby, enhancing molybdenum enzyme activity and increasing UA levels within tumors can further activate T‐cell and enhance anti‐tumor immunotherapy. To achieve this goal, biodegradable molybdenum sulfide nanoparticles (MoS_X_ NPs) were synthesized to increase molybdenum enzyme activity and thus effectively potentiate anti‐tumor immunity by integrating molybdenum‐based metalloimmunotherapy with hydrogen sulfide (H_2_S) gas therapy. This dual‐modality approach not only amplified immune activation but also triggered the stimulator of interferon genes (STING) signaling pathway and modulated purine metabolic networks, thereby orchestrating a comprehensive enhancement of anti‐tumor immune responses. In detail, the biodegradable MoS_X_ NPs exhibited excellent GSH‐responsiveness, triggering the rapid release of H_2_S and molybdate ions (MoO_4_^2−^). H_2_S‐mediated mitochondrial damage elicited the release of mitochondrial DNA (mtDNA), which activated the STING pathway, while MoO_4_^2−^ further enhanced the activation of the cGAS‐STING signaling pathway. As the catalytic moiety of molybdenum, MoO_4_^2−^ regulated cellular purine metabolic reprogramming and increased UA level with the tumor, thereby achieving synergistic anti‐tumor immune responses. This study proposes a molybdenum‐based nanocatalytic strategy to improve purine metabolic networks, activate T‐cell, and trigger a strong anti‐tumor response, thereby achieving precision metabolic‐immune therapy for tumors.

## Graphical abstract

Biodegradable molybdenum sulfide nanoparticles were synthesized via a one‐pot strategy to increase molybdenum enzyme activity and thus effectively potentiate anti‐tumor immunity by integrating molybdenum‐based metalloimmunotherapy with hydrogen sulfide gas therapy.

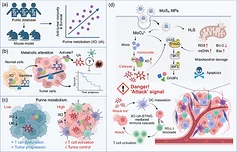

## 1 Introduction

Molybdenum (Mo) is an essential trace element in the human body that acts as the catalytic site for molybdoenzymes such as xanthine oxide (XO) and aldehyde oxidase (AO) [1, 2, 3, 4]. This critical role is attributed primarily to the fact that Mo exhibits variable oxidation states, a unique property that underlies its biological functions. Among these molybdoenzymes, xanthine oxidoreductase (XOR) serves as a pivotal enzyme in purine metabolism and is primarily distributed in hepatic, renal, intestinal, and vascular endothelial tissues [5]. The XOR homodimer, which belongs to the metalloflavoprotein family, consists of two ∼145 kDa subunits that exists in two interconvertible forms: XO and xanthine dehydrogenase (XDH) [6]. In purine catabolism, its catalytic core incorporates a molybdenum cofactor (Moco), two non‐identical iron‐sulfur clusters, and a flavin adenine dinucleotide (FAD) [7, 8, 9, 10]. Electrons are shuttled through these centers from substrates (hypoxanthine, xanthine, or aldehydes) to drive the oxidation reaction, yielding uric acid (UA) [11, 12]. Thus, it provides an important theoretical anchor for subsequent studies that systematically explore the regulatory mechanisms of purine metabolism and the physiological functions of UA, starting from the “Mo‐XOR‐UA axis”.

**SCHEME 1:**
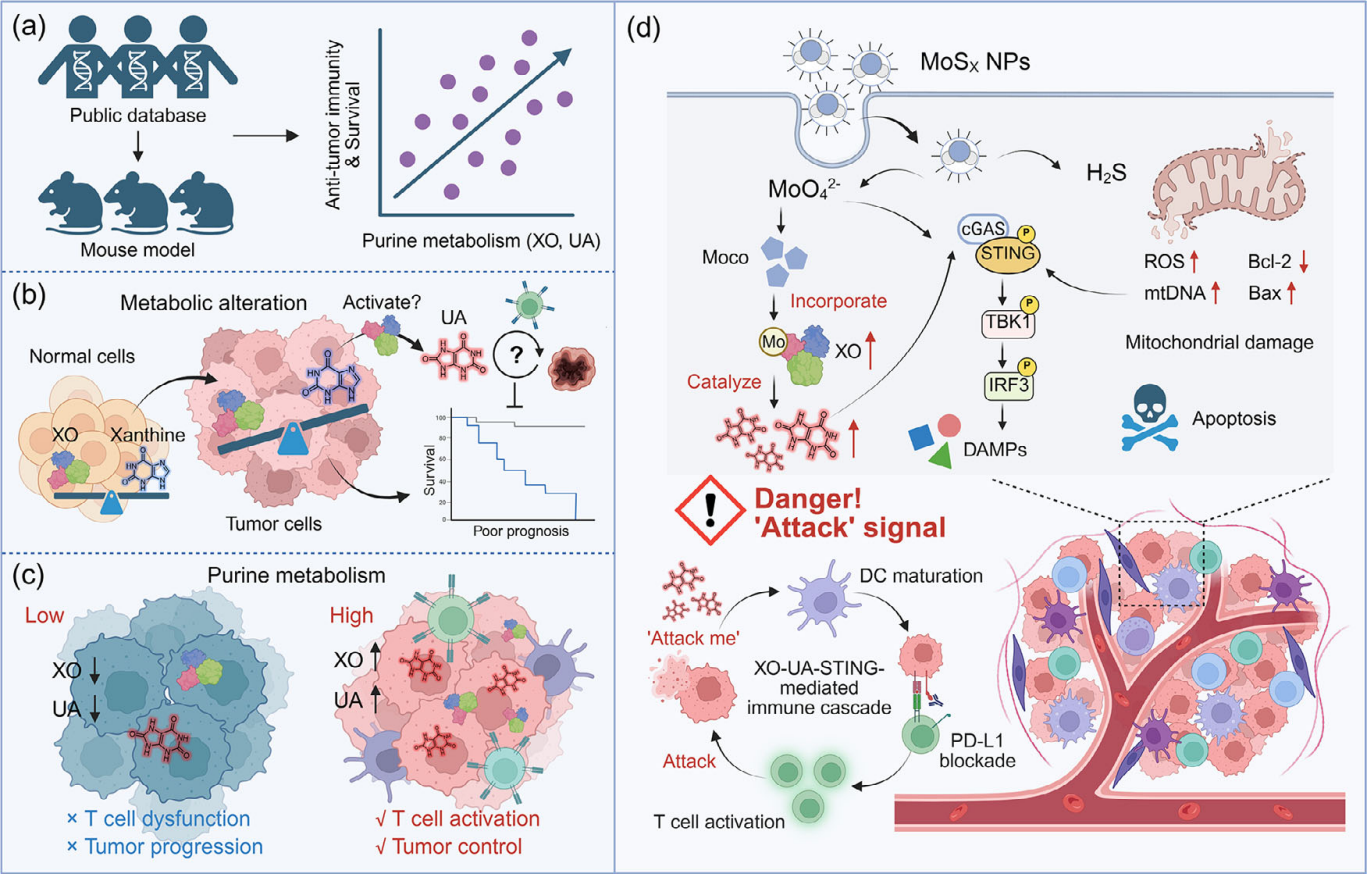
Schematic illustration of the underlying therapeutic mechanism of MoS_X_ NPs. (a) Public databases and mouse models link purine metabolism (XO, UA) to anti‐tumor immunity and survival. (b) Metabolic differences between normal and tumor cells: XO/UA in tumors associated with poor prognosis. (c) Purine metabolism (XO, UA) levels: low content drives T‐cell dysfunction and tumor progression; high content promotes T‐cell activation and tumor control. (d) MoS_X_ NPs mediate anti‐tumor immunity through XO activation, the cGAS‐STING pathway, and immune cascades (DC maturation, T‐cell activation, and PD‐L1 blockade).

UA, as the end product of purine nucleotide catabolism, can also be synthesized from ribose 5‐phosphate via a series of enzymatic reactions, primarily in the liver [13]. Under physiological conditions, UA exerts balanced pro‐ and anti‐inflammatory effects [14, 15]. For example, UA triggers NALP3 inflammasome activation, thereby leading to the release of pro‐inflammatory cytokines (e.g., IL‐1β and IL‐6) and subsequent inflammatory reactions [16]. In addition to its antioxidant properties, UA has also garnered significant attention for its immunomodulatory role, which delays the apoptosis of immune cells such as T cells, B cells, and macrophages, thereby maintaining immune defenses [17]. More evidence has revealed that UA is an immunological danger signal that stimulates dendritic cells (DCs) maturation and potentiates CD8^+^ T‐cell responses [18, 19, 20]. Thus, regulating the UA contents within tumor significantly effects anti‐tumor immune responses. Iron molybdate (FeMoO_4_), an artificial metabolic enzyme designed to mimic the tetrahedral coordination of iron and molybdenum in natural XOR, efficiently catalyzes the conversion of xanthine to UA, thereby activating the NLRP3 inflammasomes and recruiting immune cells to attack tumors [21]. Therefore, great efforts should be inputted to develop a more effective strategy for regulating molybdoenzyme activity and purine metabolic networks.

Inspired by a study that molybdenum, released during the in vivo degradation of molybdenum‐based nanomaterials, can be incorporated into the active centers of natural molybdoenzymes such as AO and XOR, which in turn enhances the specific activity of these enzymes [22]. Herein, biodegradable molybdenum sulfide NPs (MoS_X_ NPs) were synthesized via a one‐pot strategy to regulate molybdenum enzyme activity and increase UA levels within tumors (Scheme 1). The biodegradable MoS_X_ NPs triggering the rapid release of H_2_S and molybdate ions (MoO_4_^2−^). H_2_S‐mediated mitochondrial damage elicited the release of mitochondrial DNA (mtDNA), which activated the STING pathway, while MoO_4_^2−^ further enhanced the activation of the cGAS‐STING signaling pathway. As the catalytic moiety of molybdenum, MoO_4_^2−^ regulated cellular purine metabolic reprogramming and increased UA level with the tumor, thereby achieving synergistic anti‐tumor immune responses. This study highlights a molybdenum‐based nanocatalytic strategy to improve purine metabolic networks, activate T‐cell, and trigger a strong anti‐tumor response, thereby achieving tumor precision metabolic‐immune therapy. As a biocompatible molybdenum‐based platform, it circumvents the intrinsic toxicity associated with conventional metal‐based nanomaterials and exhibits favorable biosafety and metabolic profiles in vivo. By modulating tumor metabolism to revitalize effector T cells, MoS_X_ NPs effectively improve the responsiveness of tumor immunotherapy. Furthermore, its synergistic multi‐pathway regulatory mechanism helps overcome therapeutic resistance, offering a promising strategy to enhance the efficacy of cancer treatment.

## 2 Results and Discussion

### 2.1 Strong Relationship Between Purine Metabolism and Body Immunity

Initially, transcriptomic integration of cancer patient cohorts was conducted to reveal the relationship of purine metabolism with body immunity, especially T‐cell activation and infiltration. Based on the mining and analysis of clinical databases, we identified that purine metabolism‐related genes were closely associated with anti‐tumor immunity, such as immune cell functions. Principal component analysis (PCA) revealed distinct clustering and separation of patient samples in the principal component space, distinguishing the high purine metabolism group (C1) from the low purine metabolism group (C2) (Figure 1A). The heatmap visually delineated the gene expression differences between the C1 and C2 groups, thereby providing an intuitive basis for the screening and identification of key differentially expressed genes (Figure 1B). Kaplan‐Meier survival analysis combined with the log‐rank test revealed a significant statistical difference in the overall survival curves between the C1 and C2 groups, with the C1 group exhibiting a greater overall survival benefit (Figure 1C). Immune infiltration analysis revealed that samples in the C1 group presented significantly increased infiltration levels of immune cells, including plasma cells, resting CD4^+^ memory T‐cell, activated CD4^+^ memory T‐cell, and follicular helper T‐cell. This finding suggested a potentially more active tumor immune microenvironment in the C1 group (Figure 1D). Furthermore, across multiple tumor types, including melanoma, lung adenocarcinoma, hepatocellular carcinoma, and colon cancer, the expression profiles of target genes (e.g., Xdh, Pnp, and Abcg2) in CD4^+^ T cell and CD8^+^ T cell subsets were analyzed. It could be found that the expression levels of these genes exhibited significant heterogeneity across different tumor types and T‐cell subsets. Notably, the expression levels of these genes were significantly positively correlated with immune response intensity, suggesting that these purine metabolism‐related genes may be involved in the remodeling of the tumor immune microenvironment by regulating the function of T‐cell subsets (Figure 1E–H). To further confirm this phenomenon, preliminary experiments were carried out on colon cancer‐bearing mice to explore the links between immune function, T‐cell activation, XO activity, and UA content (Figure 1I). It could be found that mice with strong anti‐tumor immunity exhibited smaller tumor volumes and weights than did mice with weak anti‐tumor immunity (Figure 1J–M). After 12 days, the tumors were harvested for flow cytometry, section staining and analysis of interferon‐gamma (IFN‐γ) levels, and XO activity and UA content were assayed via commercial kits. Compared with those from the mice with weak anti‐tumor immunity, the CD3^+^ and CD8^+^ stained sections from the mice with strong anti‐tumor immunity presented stronger fluorescence (Figure 1N), increased IFN‐γ levels (Figure 1O and Figure S1), and significantly increased XO activity (Figure 1P) and UA content (Figure 1Q), indicating that the mice with strong anti‐tumor immunity exhibited more activated T‐cell and purine metabolism, thus having better anti‐tumor effects. These results collectively illustrated the strong relationships of the activity of molybdenase and UA with anti‐tumor immunity, especially T‐cell activation and infiltration (Figure 1R). Unlike existing purine metabolism‑oriented strategies, which focus mainly on inhibiting UA production for the clinical management of gout and hyperuricemia [23, 24], the present work innovatively increases UA generation to elicit antitumor immunity through its immunostimulatory activity, representing a new application of purine metabolic modulation in cancer therapy.

**FIGURE 1:**
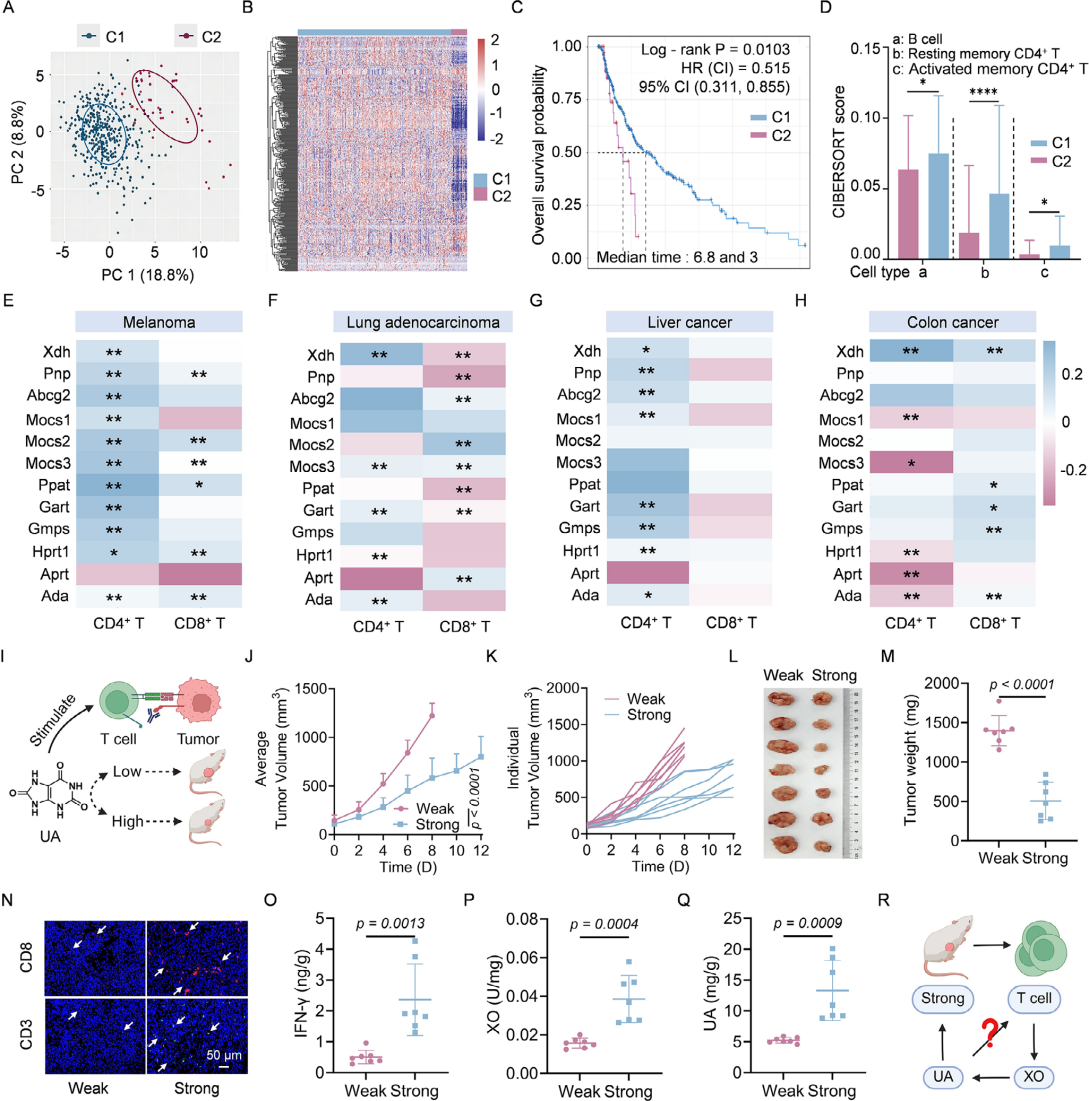
Relationships between strong or weak anti‐tumor immunity and T cells, XO, and UA. (A) PCA of TCGA melanoma samples based on purine metabolism gene expression. C1 indicates the high‐expression group, and C2 represents the low‐expression group. (B) Heatmap of purine metabolism‐related gene expression in TCGA melanoma samples. (C) Overall survival curves of C1 and C2 groups in TCGA melanoma samples. (D) Infiltration levels of immune cell subsets in TCGA melanoma samples. (E‐H) Correlation heatmap between purine metabolism genes and CD4^+^/CD8^+^ T cell infiltration in melanoma (E), lung adenocarcinoma (F), liver cancer (G), and colon cancer (H). (I) Schematic diagram of strong or weak anti‐tumor immunity. (J, K) Average tumor growth curves (J) and individual tumor growth curves (K) of CT26 tumor‐bearing mice. (L, M) Photo images (L) of tumor sizes and tumor weights (M) in mice with strong or weak anti‐tumor immunity. (N) Confocal imaging of CD3^+^ and CD8^+^ stained tumor sections. (O‐Q) The levels of IFN‐γ (O), XO activity (P) and UA content (Q) in mice with strong or weak anti‐tumor immunity. (R) Schematic diagram of the relationships among the immune system, T cells, and purine metabolism. n.s.: p > 0.05, *p < 0.05, **p < 0.01, ***p < 0.001, and the data are presented as the mean ± SD.

### 2.2 Synthesis and Characterization of MoS_X_ Nanoparticles

Given the key role of molybdoenzymes in anti‐tumor immunity and the biological process by which molybdenum (Mo) integrates into molybdoenzymes to increase their activity, the construction of a potential molybdenum donor is warranted. Molybdenum disulfide (MoS_2_), a typical 2D material, has become a hotspot in nanomaterial research because of its unique electrical, mechanical, and chemical properties. Ultra‐small MoS_X_ NPs were synthesized via a one‐pot method, where molybdenum pentachloride (MoCl_5_), sodium hydrosulfide (NaHS), and polyvinylpyrrolidone (PVP) were stirred at room temperature for 4 h (Figure 2A). Transmission electron microscopy (TEM) images and particle size distribution (PSD) histograms revealed that the ultrasmall MoS_X_ NPs exhibited a uniform structure with an average diameter of ∼ 5.7 nm (Figure 2B and Figure S2A). Based on high‐resolution TEM, the lattice spacing was measured to be approximately 0.193 nm (Figure S2B), indicating the good crystal structure of the MoS_X_ NPs. Moreover, energy‐dispersive X‐ray spectroscopy (EDS) revealed that Mo and S were uniformly distributed in the MoS_X_ NPs (Figure 2C). X‐ray photoelectron spectroscopy (XPS) analysis of the Mo 3d spectrum revealed the coexistence of Mo with multiple valence states (Mo^4+^ (binding energy, BE, ca. 240.48 and 234.08 eV), Mo^5+^ (BE ca. 234.88 and 230.98 eV), and Mo^6+^ (BE ca. 236.78 and 231.78 eV)), and their ratios were analyzed to be 33%: 9%: 58%, indicating that the value of ‘X’ was calculated to be approximately 2.83 (Figure 2D and Figure S2C). Moreover, the XPS of S2p also demonstrated the existence of sulfur deficiency in the MoS_X_ NPs (Figure 2E). As revealed by infrared (IR) spectroscopy, two characteristic peaks at ∼625 cm^−^^1^ and 940 cm^−^^1^ were attributed to the stretching vibrations of Mo‐S and S‐S, respectively, indicating the successful preparation of MoS_X_ NPs (Figure S2D). Thermogravimetric analysis (TGA) revealed that ∼28% of the PVP polymer was inserted into the MoS_X_ NPs, ensuring their excellent stability and dispersion in physiological environments like saline solution (0.9% NaCl), phosphate buffered saline (PBS), and RPMI 1640 cell medium (Figure 2F).

**FIGURE 2:**
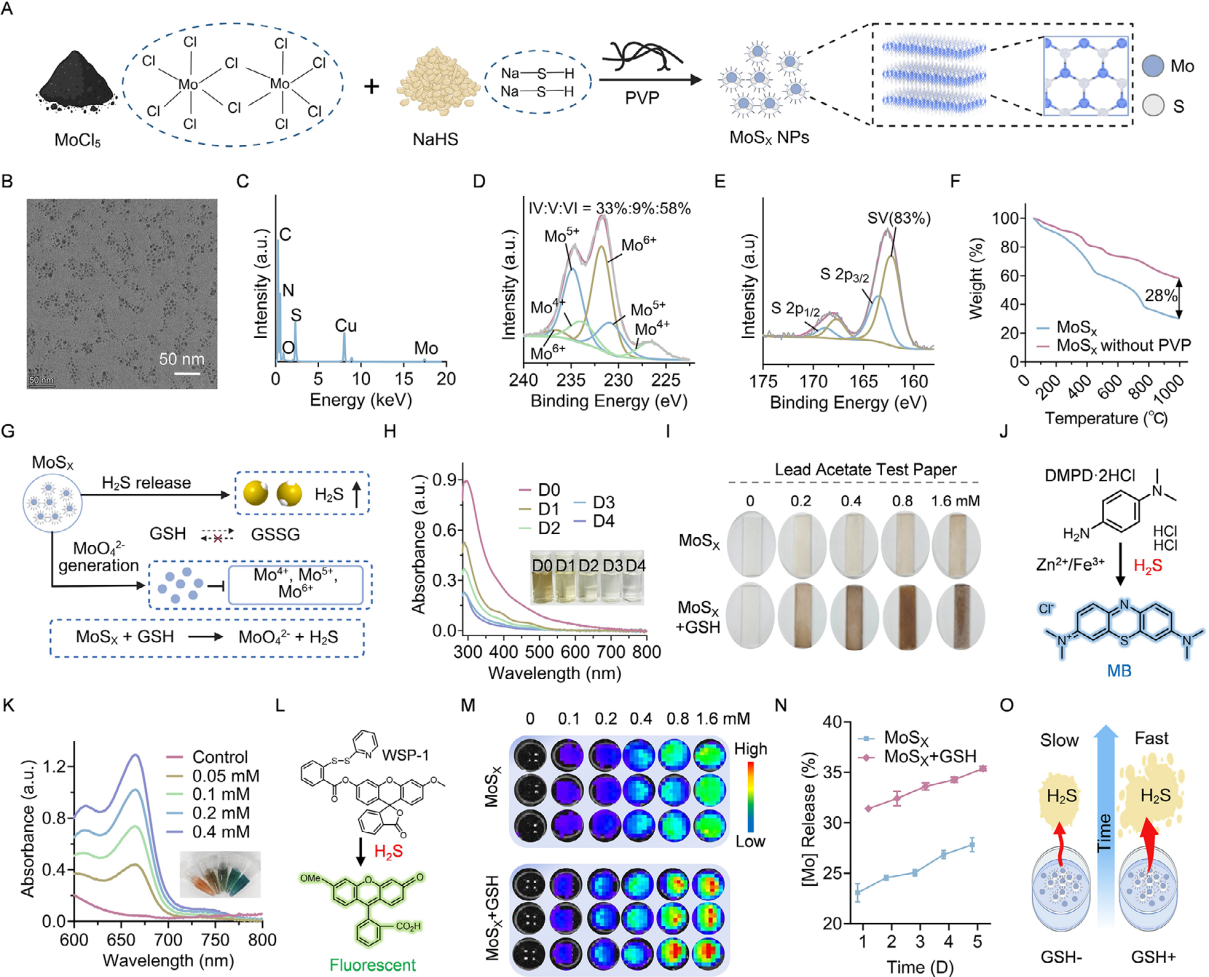
Synthesis, characterization and degradation performance of the MoS_X_ NPs. (A) Schematic illustration of the synthesis process of MoS_X_ NPs. (B) TEM images of MoS_X_ NPs. (C) EDS spectrum of MoS_X_ NPs. (D) XPS spectrum of the Mo 3d peak. (E) XPS spectrum of the S 2p peak. (F) TGA of MoS_X_ NPs before and after PVP modification. (G) Schematic diagram of MoS_X_ NPs degradation. (H) UV‐Vis‐NIR absorption spectra of MoS_X_ NPs in PBS from day 0 to day 4. (I) GSH‐responsive H_2_S release assay using the lead acetate method. (J) Schematic diagram of the principle for detecting H_2_S via the zinc acetate‐methylene blue method. (K) Absorption spectra of the probe after incubation with different concentrations of MoS_X_ NPs. (L) Schematic diagram of the principle for detecting H_2_S using the WSP‐1 probe. (M) Fluorescence images of the WSP‐1 probe in the presence or absence of GSH with MoS_X_ NPs. (N) Release curve of molybdenum ions from day 1 to day 5. (O) Schematic diagram of the rate of H_2_S release from MoS_X_ NPs in the presence or absence of GSH.

### 2.3 Degradation Performance of MoS_X_ for Molybdate and H_2_S Release

With abundant Mo and S, the MoS_X_ NPs undergo the following reaction (MoS_X_ + GSH

MoO_4_^2−^ + H_2_S) to efficiently generate H_2_S gas and MoO_4_^2−^ (Figure 2G). Subsequently, the biodegradability of them was investigated in detail. After incubation in PBS at 37°C, the color of the MoS_X_ solution changed from pale yellow to colorless, and the UV‐vis absorption sharply decreased (Figure 2H). Then, the degradation products (H_2_S and MoO_4_^2−^) were monitored. First, lead acetate test paper (lead acetate, Pb(CH_3_COO)_2_, which can react with H_2_S to form black PbS precipitates) exhibited progressively darker staining after incubation with MoS_X_ NPs at increasing concentrations, indicating the efficient release of H_2_S (Figure 2I and Figure S3). Next, methylene blue (MB) spectrophotometry (H_2_S reacts with Zn^2+^ to form a ZnS precipitate, while in an acidic solution containing Fe^3+^, it reacts with *N, N*‐dimethyl‐p‐phenylenediamine hydrochloride (DMPD·2HCl) to generate MB) revealed that the UV‐vis absorption of the probe at ∼665 nm increased sharply following incubation with varying concentrations of MoS_X_ NPs, accompanied by a color transition from brown to dark green (Figure 2J, K). Finally, WSP‐1 serves as a fluorescent probe for H_2_S, rapidly reacting with H_2_S to yield phenylpropanedithiol ketone with a strong fluorescent signal (Ex = 465 nm, Em = 515 nm) (Figure 2L). In vivo fluorescence imaging revealed that the fluorescence intensity progressively increased with enhancing concentrations of MoS_X_ NPs (Figure 2M). Meanwhile, we also observed significant release of MoO_4_^2−^ from the MoS_X_ NPs, further indicating the biodegradability of the MoS_X_ NPs for efficient generation of H_2_S and MoO_4_^2−^ (Figure 2N). Given that high levels of glutathione (GSH) and weak acidity are widely reported in the tumor microenvironment (TME), we hypothesized that the degradation of MoS_X_ NPs could be promoted by these factors. As revealed by the above MB spectrophotometry method, lead acetate test paper, and WSP‐1 probe, H_2_S release was significantly accelerated (Figure 2I, M and Figure S4–S6). Inductively coupled plasma optical emission spectra (ICP‐OES) revealed the rapid release of MoO_4_^2−^. In brief, the MoS_X_ NPs themselves exhibited excellent biodegradability, and this process could be further accelerated by the presence of GSH and a low pH value in the TME (Figure 2O and Figure S7), thereby triggering the abundant release of H_2_S and MoO_4_^2−^ for biological applications.

### 2.4 Biological Function of a Degraded Product, H_2_S

Given the excellent biodegradability of the MoS_X_ NPs, we further investigated their ability to undergo intracellular degradation to further induce cancer cell death (Figure 3A). Initially, the cytotoxicity of the MoS_X_ NPs was evaluated via a standard methyl thiazolyl tetrazolium (MTT) assay. After incubation with murine colon cancer CT26 cells for 24 h, NaHS and Na_2_MoO_4_, the inorganic salt donors of H_2_S and MoO_4_^2−^, exhibited weak cytotoxicity, while the MoS_X_ NPs significantly inhibited cell proliferation (Figure 3B). This was attributed to the precise acidity/GSH‐responsive H_2_S and MoO_4_^2−^ release inside the cancer cells, avoiding the “off‐target” effect, in contrast with the uncontrolled release of inorganic salts. Similar results were observed in murine breast cancer 4T1 cells, but this cytotoxicity disappeared in normal cells (human umbilical vein endothelial cells (HUVECs) and mouse dendritic cells (DC2.4) (Figure 3C). This phenomenon was attributed mainly to the fact that low GSH and normal pH values were not conducive to the intracellular decomposition of MoS_X_ NPs. Moreover, these compounds also exhibited time‐dependent cytotoxicity, indicating that their continuous degradation induces long‐term cell killing effects inside cancer cells (Figure 3D). Next, intracellular H_2_S was monitored by the WSP‐1 probe, with the thiol scavenger *N*‐ethylmaleimide (NEM) as a negative control and NaHS as a positive control. Compared with those in the control and MoO_4_^2−^ groups, weak green fluorescence was observed in the NaHS group, and a strong fluorescent signal appeared in the MoS_X_ group, indicating their precise and efficient decomposition of them inside the cancer cells (Figure 3E, K). However, after co‐treatment with NEM, no fluorescent signal was detected, indicating efficient scavenging of the intracellular thiol. The decomposition process could consume the intracellular GSH, thereby disrupting the intracellular redox balance. Thus, the intracellular GSH levels were detected by Thiol Tracker Violet (Figure 3I). Apparently, the GSH level inside CT26 cells after MoS_X_ treatment significantly decreased, and this phenomenon aggravated with increasing concentrations of MoS_X_ NPs. The quantitative results of the GSH/GSSG detection kits also confirmed the significant decrease in intracellular GSH after MoS_X_ treatment (Figure 3N).

**FIGURE 3:**
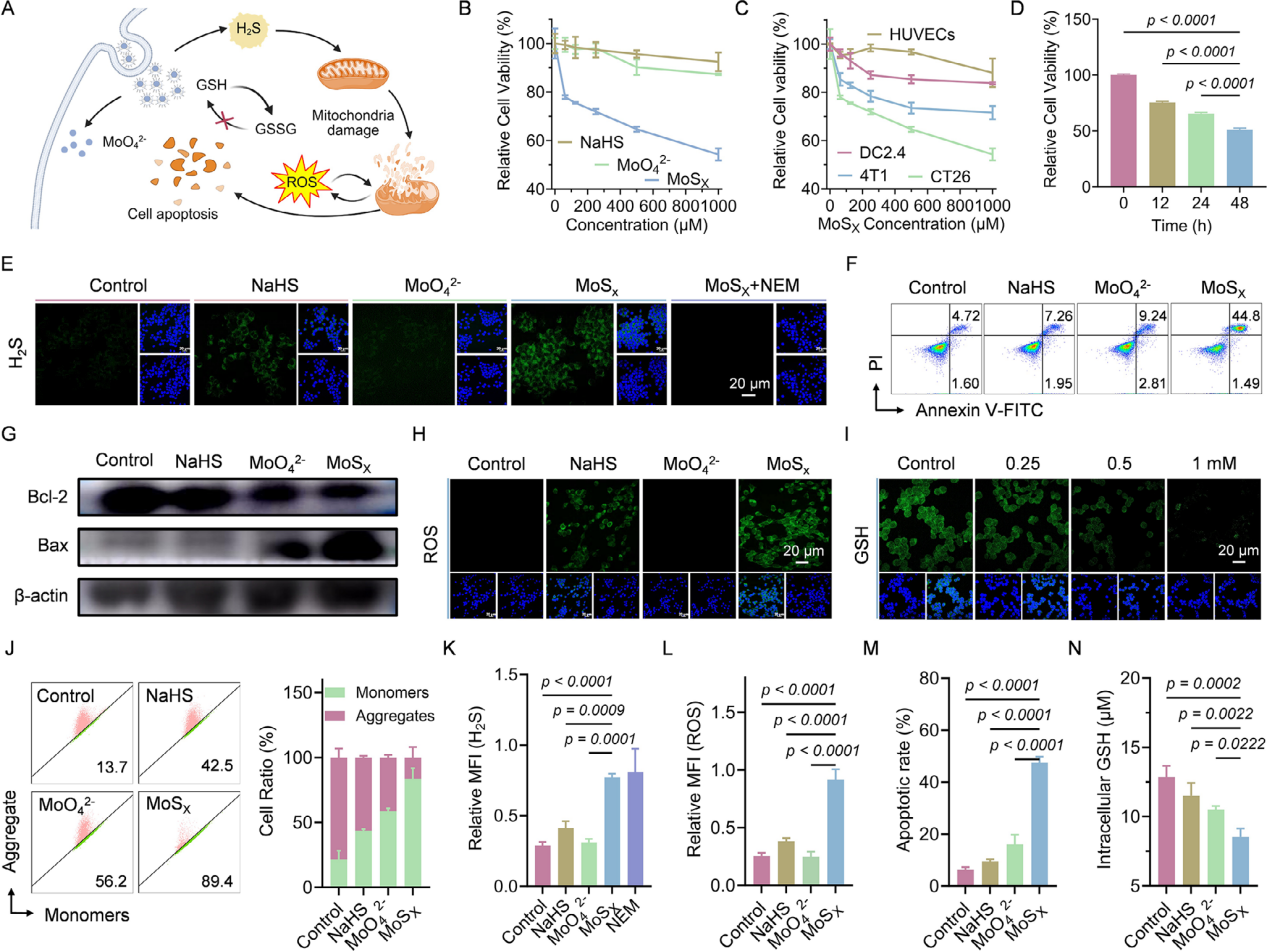
In vitro MoS_X_ induces apoptosis. (A) Schematic diagram of the biological effects of MoS_X_ in cells. (B) Relative survival of CT26 cells after 12 h of treatment with NaHS, MoO_4_^2−^, or MoS_X_. (C) Relative survival of HUVECs, DC2.4, 4T1, and CT26 cells after MoS_X_ treatment. (D) Relative viability of CT26 cells treated with MoS_X_ for different durations. (E) Fluorescence staining of intracellular H_2_S in CT26 cells after different treatments. (F) Flow cytometric analysis of cell apoptosis after different treatments. (G) Western blot analysis of apoptosis pathway‐related proteins. (H) ROS fluorescence staining of CT26 cells after different treatments. (I) GSH fluorescence staining of CT26 cells after different treatments. (J) Flow cytometry and quantitative analysis of JC‐1 after different treatments. (K, L) Fluorescence quantification of H_2_S (K) and ROS (L) levels. (M) Fluorescence quantification of the percentage of apoptotic cells. (N) Intracellular GSH levels in CT26 cells. n.s.: p > 0.05, *p < 0.05, **p < 0.01, ***p < 0.001, and the data are presented as the mean ± SD.

Excess intracellular H_2_S interferes with the mitochondrial respiratory chain, thus damaging mitochondrial function and inducing cell death [25, 26]. Subsequently, JC‐1, a mitochondrial membrane potential (MMP) indicator, was used to evaluate the interference of MoS_X_ NPs on mitochondrial activity. Red fluorescence of JC‐1 aggregates (normal mitochondria) was clearly observed in the control group, and weak green fluorescence of JC‐1 monomers (abnormal mitochondria) appeared in the NaHS and MoO_4_^2−^ groups, indicating their partial inhibition of mitochondrial function. In particular, the MoS_X_ group exhibited strong green fluorescence, revealing severe damage to mitochondrial function (Figure S8). Flow cytometry analysis also verified these results, which may be attributed to the precise and efficient decomposition of the MoS_X_ NPs to generate H_2_S inside the cancer cells (Figure 3J). Mitochondria are recognized as one of the primary sources of reactive oxygen species (ROS) production, with increasing ROS levels upon mitochondrial damage [27, 28]. Thus, owing to the significant damage to mitochondria caused by MoS_X_ NPs, the intracellular ROS levels were further investigated via the 2',7'‐dichlorofluorescin diacetate (DCFH‐DA) fluorescent probe (Figure 3H, L). Notably, CT26 cells treated with MoS_X_ NPs exhibited strong green fluorescence, indicating that high levels of ROS were generated in these cells. All these results were attributed, on the one hand, to intracellular H_2_S‐induced mitochondrial dysfunction, and on the other hand, to GSH depletion and redox imbalance caused by MoS_X_ decomposition (Figure S9). The high oxidative stress was strongly cytotoxic and induced significant cell apoptosis.

The cytotoxicity was further confirmed by the Annexin V‐FITC/PI assay (Figure 3F, M), and the results were consistent with the trend observed in the MTT assay. Stimulation of apoptotic signals leads to changes in the expression of mitochondrial apoptosis‐related proteins [29, 30], including anti‐apoptotic proteins such as B‐cell lymphoma‐2 (Bcl‐2) and pro‐apoptotic proteins such as BCL2‐Associated X (Bax) (Figure 3G). As revealed by western blot (WB) analysis, the MoS_X_‐treated CT26 cells showed downregulated Bcl‐2 expression and upregulated Bax expression, further confirming the significant degree of apoptosis induced by the MoS_X_ NPs. Collectively, the degradation product H_2_S caused mitochondrial damage, amplified oxidative stress, and ultimately induced significant cell apoptosis.

### 2.5 Biological Function of Another Degraded Product, MoO_4_^2−^

Apart from H_2_S, the biological functions of another degradation product, MoO_4_^2−^, were also investigated. As one of the most important types of antigen‐presenting cells (APCs), the maturation of DC cells is a vital initiator of immunity [31]. It has been reported that some metal ions can stimulate DC maturation and initiate immune reactions [32, 33]. To investigate whether the MoO_4_^2−^ released from MoS_X_ degradation could stimulate DC maturation, we incubated them with primary mouse bone marrow‐derived DCs (BMDCs) (Figure 4A). It could be found that both MoO_4_^2−^ and NaHS had partial stimulatory effects on DC maturation, while MoS_X_ NPs exhibited the strongest stimulatory effects, comparable to those of lipopolysaccharide (LPS) (Figure 4B, C). These results might be attributed to the increase in the amount of intracellular MoO_4_^2−^ after the uptake of MoS_X_ NPs by the cells.

**FIGURE 4:**
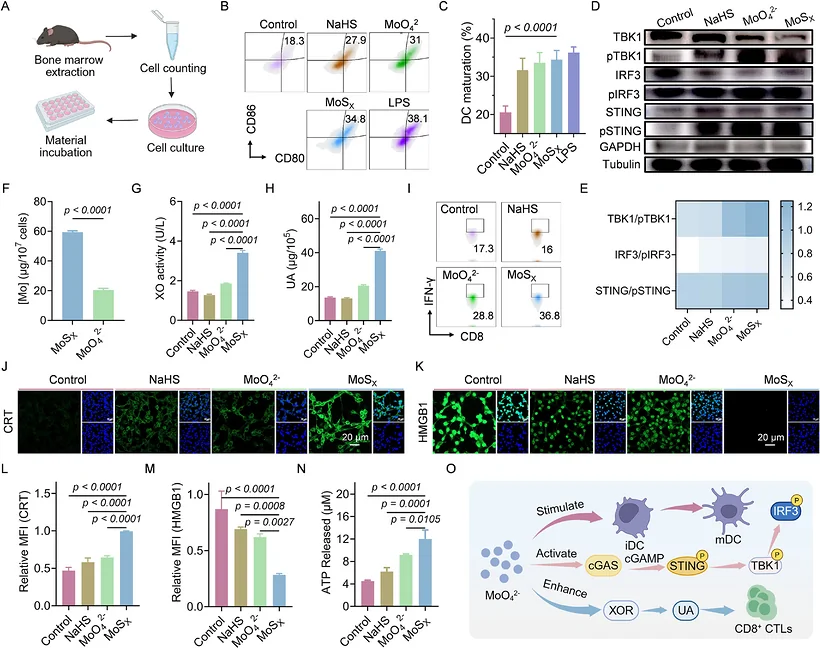
In vitro MoO_4_^2−^ induces DC maturation, activates the cGAS‐STING pathway, and increases XO activity and UA content. (A) Diagram of the cultivation of mouse bone marrow‐derived dendritic cells. (B, C) Flow cytometric detection and quantification of BMDCs maturation. (D, E) Western blot analysis and quantification of cGAS‐STING pathway‐related proteins. (F) Determination of the intracellular Mo content. (G, H) Assay of intracellular XO activity (G) and UA content (H). (I) Flow cytometric analysis of the activated T cells. (J, K) CRT and HMGB1 staining of tumor sections. (L, M) Quantitative fluorescence analysis of CRT and HMGB1 staining. (N) ATP detection after different treatments. (O) Schematic diagram of MoO_4_^2−^ functions. n.s.: p > 0.05, *p < 0.05, **p < 0.01, ***p < 0.001, and the data are presented as the mean ± SD.

In addition to DC maturation, MoO_4_^2−^ has been reported to activate the cyclic GMP‐AMP synthase (cGAS)/stimulator of interferon genes (STING) pathway [34, 35, 36], suggesting its potential as an immune activation agent. To assess cGAS‐STING pathway activation by MoS_X_ NPs, the marker proteins like STING, TBK1, and IRF3 were investigated by WB analysis (Figure 4D, E). Compared with that in the control group, the phosphorylation level of STING in the MoS_X_‐treated group was significantly greater, indicating that the STING pathway was successfully activated. This STING activation subsequently recruited TBK1 and induced downstream substrate IRF3 phosphorylation, which then translocated into the nucleus and initiated the expression of type I interferons (IFN‐β) and other immune‐related genes, thereby triggering the innate immune response. Compared with the control group, NaHS‐ or MoO_4_^2−^‐treated cells showed partial increases in the phosphorylation levels of these proteins, likely due to H_2_S‐induced mitochondrial damage, which releases mtDNA to bind cGAS and activate STING [37, 38]. In contrast, the protein phosphorylation levels in the MoS_X_ group were most significantly elevated, which was attributed to the positive effects of mtDNA and intracellular MoO_4_^2−^ on the activation of the cGAS‐STING pathway. Unlike conventional STING activating systems that act through single pathway targeting, the MoS_X_ system activates antitumor immune responses in a multidimensional manner via the synergy of gas therapy and metal immunotherapy, leading to significantly enhanced therapeutic efficacy and tumor specificity.

Given that Mo is involved in critical biochemical reactions and that XO (a representative molybdenum enzyme) regulates purine metabolism by catalyzing the sequential conversion of hypoxanthine to xanthine and then to UA, UA enhances the antitumor activity of CD8^+^ T cells through a signaling cascade [13, 16]. Therefore, due to the degradation of MoS_X_ NPs for an efficient supply of Mo elements (Figure 4F), we hypothesized that the degradation of MoS_X_ NPs could enhance XO activity and UA production to activate T cells. As determined by commercial kits, both the XO activity and UA levels in MoS_X_‐treated cells were increased by 2‐fold compared with those in control groups (Figure 4G, H), indicating that an efficient supply of Mo element is beneficial for purine metabolism. Then, we evaluated the activation of naive T‐cell after incubation with MoS_X_ NPs (Figure 4I). Compared with those in the control group, the level of MoO_4_^2−^, as a molybdenum source, partially activated T‐cell, and the T‐cell activation rate in the MoS_X_ group was the highest levels among these four groups, indicating that the MoS_X_ NPs significantly increased the intracellular Mo level, accelerated the formation of XO, further promoted the production of UA and subsequently activated T‐cell.

Immunogenic cell death (ICD) is characterized by the release of damage‐associated molecular patterns (DAMPs) from dying tumor cells, which activate antitumor immune responses [39]. Key DAMPs include cell surface‐exposed calreticulin (CRT), secreted high‐mobility group box 1 protein (HMGB1), and released adenosine triphosphate (ATP), which serve as canonical ICD markers [40, 41]. Given the critical biological functions of the degradation products of MoS_X_ NPs (H_2_S and MoO_4_^2−^), we aimed to evaluate the ICD effect and detect DAMPs from cancer cells following MoS_X_ NPs treatment (Figure 4J–M). Compared with control cells, MoS_X_‐treated CT26 cells exhibited significantly enhanced exposure of CRT on the cell surface, as evidenced by intense green fluorescence by confocal imaging. Regarding HMGB1 secretion, this nuclear protein is released into the extracellular space during apoptosis, whereas in MoS_X_‐treated cells, its extracellular levels are decreased. This extracellular secretion of HMGB1 enhances antigen presentation by DCs to T cells, thereby promoting immune activation. Concurrently, MoS_X_‐induced cell death was associated with a marked increase in the level of extracellular ATP, which was released from dying cells into the microenvironment (Figure 4N). Above all, the constructed bioactive MoS_X_ nanomaterial degraded into H_2_S and MoO_4_^2−^ upon entering cells, inducing apoptosis and ICD while enhancing the immunogenicity of cancer cells (Figure 4O). MoS_X_ NPs do not merely rely on the passive immunomodulatory effects of metal ions but instead exert a dual function by enhancing endogenous molybdenum enzymes activity and precisely regulating the purine metabolic pathway in the tumor microenvironment, thereby achieving synergistic metabolic reprogramming and immune activation.

### 2.6 In Vivo Antitumor Efficacy of MoS_X_ NPs

Building on the promising biological functions of H_2_S and MoO_4_^2−^, we evaluated the antitumor activity of MoS_X_ in a CT26 tumor model. Mice bearing CT26 tumors were divided into four groups (n = 5 per group): 1) control; 2) NaHS (intratumoral (i.t.) injection, 40 mM, 50 µL); 3) MoO_4_^2−^ (i.t. injection, 20 mM, 50 µL); and 4) MoS_X_ (i.t. injection, 20 mM, 50 µL) (Figure 5A). MoS_X_ NPs were injected at days 0, 2, or 4 for a total of three injections, NaHS and MoO_4_^2−^ were treated at the same injection schedule as MoS_X_ NPs, and these tumor sizes were monitored every two days. Compared with those in the control group, the growth of tumors in the NaHS and MoO_4_^2−^ groups was partially inhibited (Figure 5B, E). These results may be attributed to the fact that H_2_S disrupts mitochondrial ATP synthesis and inhibits tumor cell proliferation, whereas MoO_4_^2−^ activates the STING signaling cascade to induce the release of type I interferons and trigger immune‐mediated tumor growth inhibition. More importantly, the MoS_X_‐injected tumors were more significantly inhibited than the other three groups (Figure 5C), further indicating that MoS_X_ exerts dual antitumor effects not only by releasing H_2_S to induce tumor cell death but also by generating MoO_4_^2−^ to activate the STING pathway, promote immune cell activation, and modulate purine metabolism. The survival duration of the mice in the MoS_X_ group was significantly longer than that of the other groups (Figure 5D). As revealed by hematoxylin and eosin (H&E) staining (Figure 5F), minimal nuclear condensation and modest cell loss were observed in the NaHS and MoO_4_^2−^ groups, while in the MoS_X_‐treated group, the cells were small, rounded, with sparse cytoplasm and indistinct boundaries, accompanied by extensive nuclear condensation and apoptosis, reflecting severe cellular damage. As a validated indicator of tumor cell proliferative capacity (Figure 5F, G), Ki67 expression analysis revealed that the MoS_X_‐treated group exhibited the lowest levels compared with those of the other experimental groups, further indicating the great therapeutic efficacy of the MoS_X_ nanomaterials. Owing to its efficient degradation and abundant supply of H_2_S and MoO_4_^2−^, MoS_X_ exerted multiple roles, such as inhibiting cell metabolism, activating the cGAS‐STING pathway, and triggering an anti‐tumor immune response, all of which are favorable for efficient antitumor therapy.

**FIGURE 5:**
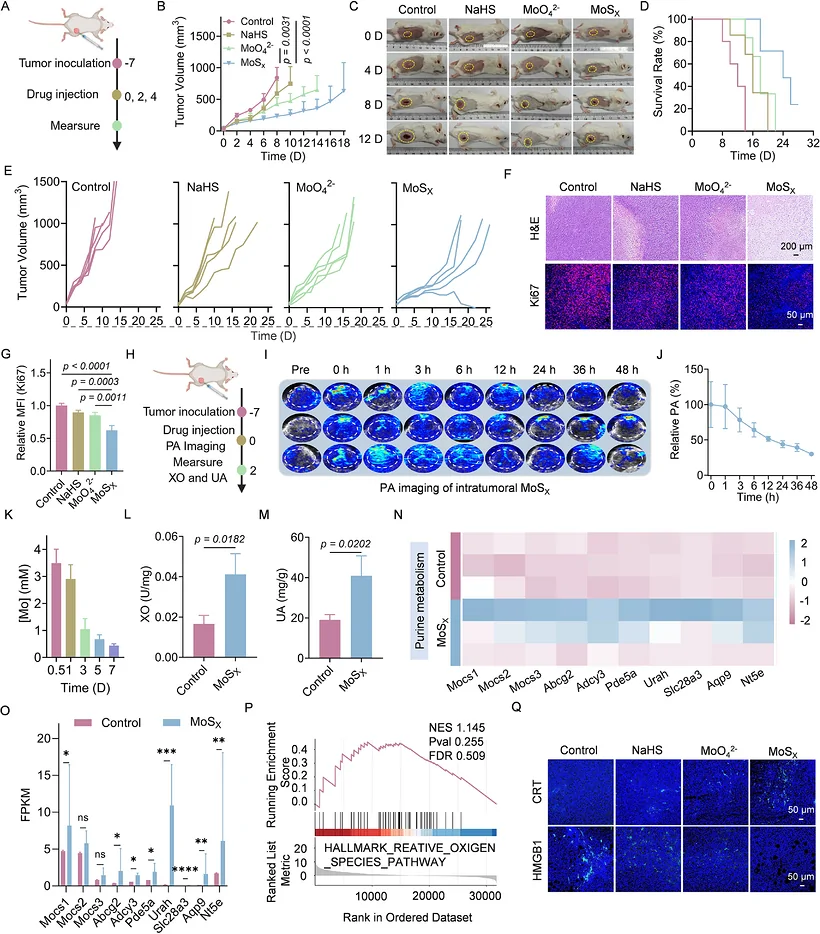
In vivo antitumor effects of the MoS_X_ NPs. (A) MoS_X_ treatment plan for CT26 tumor‐bearing mice. (B) Average tumor growth curves of CT26 tumor‐bearing mice after different treatments. (C) Tumor images of tumor‐bearing mice in different treatment groups at various time points. (D) Survival curves of the mice after treatment. (E) Individual tumor growth curves of CT26 tumor‐bearing mice after different treatments. (F) H&E and Ki67 stained images of tumor sections. (G) Fluorescence quantification of Ki67 expression. (H) Schematic diagram of MoS_X_ photoacoustic imaging and XO/UA measurement. (I, J) PA imaging (I) and statistical analysis (J) of mice injected with MoS_X_ at different times. (K) Mo content in CT26 tumor‐bearing mice at different time points. (L, M) XO levels (L) and UA levels (M) in tumor tissues. (N) Heatmap showing up‐regulated genes involved in purine metabolism. (O) Box plot of gene expression levels at different groups. (P) GSEA analysis of purine metabolism‐related gene sets. (Q) CRT and HMGB1 staining of CT26 tumor sections from different groups. n.s.: p > 0.05, *p < 0.05, **p < 0.01, ***p < 0.001, and the data are presented as the mean ± SD.

Considering the ability of MoS_X_ NPs to enhance XO activity and UA production in vitro, we further investigated their metabolic effects in vivo via a subcutaneous CT26 tumor model (Figure 5H). Initially, photoacoustic (PA) imaging was employed to monitor the retention of MoS_X_ nanomaterials after i.t. injection (Figure 5I, J), and the results revealed a gradual decrease in the PA signal. After 2 days, the retention of the PA signal decreased to approximately 30%, indicating efficient intratumoral degradation of the MoS_X_ NPs. In addition, the intratumoral molybdenum levels decreased in a time‐dependent manner (Figure 5K). Even at 7 days, ∼ 10.5% of the Mo was still retained, suggesting that long‐term Mo retention is associated with systemic molybdoenzyme metabolism. Finally, the activity of XO and the content of UA inside the tumors were detected via commercial kits (Figure 5L, M). Compared with those in the control group, both XO activity and UA content in the MoS_X_ group were increased 2‐fold, further confirming that MoS_X_ is positively involved in molybdenum enzyme‐mediated processes and UA metabolism in vivo. To further examine this result, transcriptome sequencing (RNA‐seq) analysis of mouse tumor tissues from the control and MoS_X_ groups was conducted. As revealed by the heatmap and bar chart, these purine metabolism genes were consistently upregulated in the MoS_X_ group relative to the control group (Figure 5N, O). Specifically, the molybdenum‐related genes Mocs1, Mocs2, and Mocs3, which were involved in regulating Moco synthesis, were significantly upregulated after MoS_X_ treatment, accompanied by increased Moco production. This indirectly indicated active purine metabolism and increased XO activity. Moreover, Abcg2, a gene involved in purine metabolism, was also significantly upregulated. Abcg2 is closely associated with UA, functioning as a key UA transporter responsible for UA excretion [42]. Enhanced Abcg2 expression suggests elevated UA levels in mice. Concurrently, the ESGA plot results revealed significant enrichment of purine metabolism‐related pathways in the MoS_X_ treatment group (Figure 5P). All these results confirmed that MoS_X_ NPs enhanced XO activity and UA content at both the genetic and molecular levels. Compared with traditional enzyme‑mimicking nanomaterials [43, 44], its core advantage is that it not only functions through exogenous catalysis but also activates the intrinsic xanthine oxidoreductase pathway in vivo via molybdenum to reshape purine metabolism through endogenous regulation, overcoming the limitations of conventional nanozymes. In addition, given the roles of MoS_X_ with special biological functions and strong ICD effects in vitro, we evaluated the in vivo ICD effects. As revealed by confocal imaging, the MoS_X_‐treated tumors exhibited robust CRT surface exposure (strong green fluorescence) and low nuclear HMGB1 expression (weak green fluorescence) (Figure 5Q), indicating that the MoS_X_ NPs induced strong ICD effects, which were favorable for anti‐tumor immunity.

### 2.7 In Vivo MoS_X_‐Induced Antitumor Immunity

Due to their special biological functions, which include inhibiting cancer cell metabolism, activating the cGAS‐STING pathway, enhancing XO activity and UA products, and triggering strong ICD effects, the anti‐tumor immune responses triggered by MoS_X_ NPs have been characterized by flow cytometry analysis (Figure 6A). After 7 days post‐MoS_X_ administration, the tumors and tumor‐draining lymph nodes (TDLNs) were collected, homogenized to obtain the single‐cell suspension, and then stained for immunological analysis. By examining the maturation status of dendritic cells (DCs) in TDLNs (Figure 6B, C), It could be found that MoS_X_ treatment significantly increased DCs maturation. These phenomena were attributed mainly to the release of mtDNA and DAMPs, as well as the activation of the cGAS‐STING pathway, all of which benefited from MoS_X_ degradation products and contributed to robust antigen‐presenting capacity and potent host immune responses. MoS_X_ NPs system activates antitumor immune responses in a multidimensional manner via the synergy of gas therapy and metal immunotherapy, leading to significantly enhanced therapeutic efficacy and tumor specificity.

**FIGURE 6:**
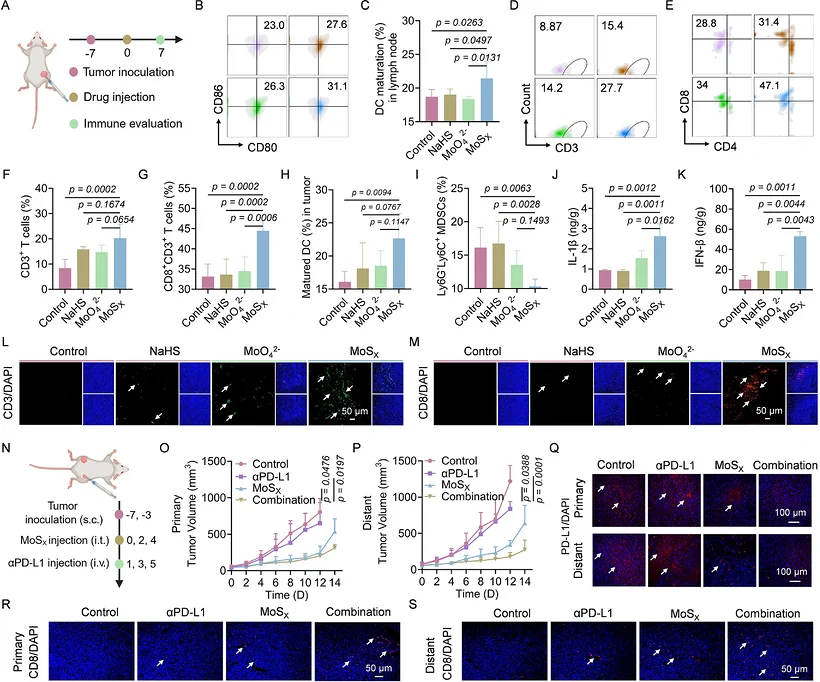
In vivo immune evaluation of the mice treated with MoS_X_ combined with αPD‐L1. (A) Schematic diagram of in vivo immune evaluation in CT26 tumor‐bearing mice. (B, C) Maturation and quantitative analysis of dendritic cells in the lymph nodes. (D) Flow cytometric analysis of CD3^+^ T cells. (E) Flow cytometric analysis of CD4^+^ and CD8^+^ T cells. (F, G) Flow cytometric quantification of total T cells (F) and CD8^+^ T cells (G). (H, I) Flow cytometric quantification of intratumoral mature dendritic cells (H) and MDSC cells (I). (J, K) Intratumoral levels of IL‐1β (J) and IFN‐β (K) from the different groups. (L, M) Staining of CD3^+^ (L) and CD8^+^ (M) cells in tumor sections. (N) Schematic diagram of the use of MoS_X_ combined with αPD‐L1 for treating CT26 tumor‐bearing mice. (O, P) Primary tumor volume (O) and distant tumor volume (P) in the mice. (Q‐S) αPD‐L1 and CD8^+^ staining of tumor sections. n.s.: p > 0.05, *p < 0.05, **p < 0.01, ***p < 0.001, and the data are presented as the mean ± SD.

In addition, the infiltration of T cells was significantly increased in the MoS_X_ group (Figure 6D–G). Compared with that in the control group, the percentage of CD3^+^ T cells increased from ∼8.87% to ∼27.7%, while the percentage of CD8^+^ CTLs increased from ∼28.8% to ∼47.1%. The proportion of DCs in tumors in the MoS_X_‐treated group was significantly increased compared with that in the control group (Figure 6H). Meanwhile, the number of monocyte‐like myeloid‐derived suppressor cells (M‐MDSCs) significantly reduced (Figure 6I), which relieved tumor immunosuppression and enhanced T‐cell proliferation, activation, and cytotoxic function [45], thereby improving tumor recognition and elimination. High levels of pro‐inflammatory cytokines, including interleukin‐1β (IL‐1β) and interferon‐β (IFN‐β), were also detected in the MoS_X_ group (Figure 6J, K). IL‐1β potently amplified inflammatory responses, while IFN‐β generated via STING pathway‐mediated IRF3 phosphorylation orchestrated immune effector functions. To further validate T‐cell activation in the MoS_X_ group, we further stained these intratumoral T‐cell and observed them via confocal imaging (Figure 6L, M). Obvious fluorescent signals (CD3, green; CD8, red) appeared in tumor tissue sections from the MoS_X_‐treated group, accompanied by markedly increased cellular expression of these markers, further confirming that MoS_X_ promotes T‐cell activation and enhances their recognition of tumor‐associated abnormal antigens, thereby mediating anti‐tumor activity. Owing to their special biological functions, MoS_X_ exerted favorable immunomodulatory effects post‐treatment, primarily through strengthening anti‐tumor immune responses and promoting the release of pro‐inflammatory cytokines.

PD‐L1 overexpression in tumor cells mediates immune evasion by binding to PD‐1 on T cells, abrogating tumor recognition [46]. Thus, an anti‐PD‐L1 antibody (αPD‐L1) was employed to address this checkpoint blockade process and enhance MoS_X_‐mediated anti‐tumor immune responses. A bilateral CT26 tumor‐bearing mouse model was established by inoculating CT26 cells on day 0 (primary tumor, left side) and day 4 (distant tumor, right side) to evaluate systemic antitumor efficacy (Figure 6N). Once the left‐sided primary tumor reached a volume of ∼80 mm^3^, the mice were randomly divided into four groups (n = 4 per group): (1) control; (2) αPD‐L1 (intravenous injection (i.v. injection), 1 mg/kg); (3) MoS_X_ (i.t. injection, 20 mM, 50 µL); and (4) combination therapy (αPD‐L1 + MoS_X_). For groups 3 and 4, MoS_X_ NPs were intratumorally injected three times on days 0, 2, and 4. Meanwhile, groups 2 and 4 were intravenously administered αPD‐L1 on days 1, 3, and 5 for a total of three times. As expected, the combination therapy with MoS_X_ and αPD‐L1 significantly inhibited primary tumor growth (Figure 6O and Figure S10A), demonstrating that MoS_X_‐mediated antitumor immune responses could enhance the effectiveness of αPD‐L1 immunotherapy. In addition, the combination therapy also notably suppressed the growth of abscopal metastatic tumors (Figure 6P and Figure S10B), while this effect was not detected with αPD‐L1 monotherapy, highlighting that MoS_X_‐mediated antitumor immune responses combined with αPD‐L1 immunotherapy could efficiently inhibit tumor growth and metastasis. Finally, a significant decrease in PD‐L1 expression and obvious CD8^+^ T‐cell infiltration appeared in bilateral tumor tissue from the combined group (Figure 6Q–S), further confirming that MoS_X_ NPs enhance XO activity and UA content for T‐cell activation and that infiltration could assist immune checkpoint blockade (ICB) immunotherapy.

Biological safety is a critical prerequisite for the application of nanomaterials in biomedicine. In this study, we systematically evaluated the potential in vivo toxicity of MoS_X_ NPs in mice through serum biochemical assays, complete blood count analysis, and histopathological examination. The results revealed that there were no significant differences in serum biochemical and hematological parameters between the MoS_X_‐treated group and the blank control group (Figures S11 and S12). Histopathological analysis further revealed no obvious tissue damage or pathological abnormalities in the major organs of the mice after MoS_X_ NPs administration (Figure S13). In conclusion, the synthesized MoS_X_ NPs did not induce significant long‐term toxic side effects in mice and exhibited favorable biosafety, laying an important foundation for their further application in the biomedical field.

### 2.8 Exploration of Potential Mechanisms

To further confirm that MoS_X_ activates T cells and participates in purine metabolism, transcriptome sequencing (RNA‐Seq) was performed on mouse tumor tissues following MoS_X_ treatment (Figure S14). Compared with those in the control group, the volcano plot revealed 4563 differentially expressed genes in the MoS_X_ group, among which 426 were downregulated and 4137 were upregulated (Figure 7A). Gene Ontology (GO) and Kyoto Encyclopedia of Genes and Genomes (KEGG) enrichment analyses revealed that the T‐cell pathways (e.g., T‐cell receptor (TCR) signaling pathways, toll‐like receptor signaling pathways; Th1, Th2 and Th17 cell differentiation pathways; cytokine‐cytokine receptor interaction pathways, and chemokine signaling pathways) and purine metabolism pathways (purine metabolism pathways) were significantly activated, with potential functional synergy between them (Figure 7B). Notably, these up‐regulated genes were associated with tumor cell apoptosis (e.g., Tnf, Slc7a11, and Gzme) (Figure S15A), mitochondrial damage (e.g., Mmp9, Abcg2, and Sod2) (Figure S15B), and TCR signaling pathways (e.g., Lcp2, Tespa1, and Zap70) (Figure 7E).

**FIGURE 7:**
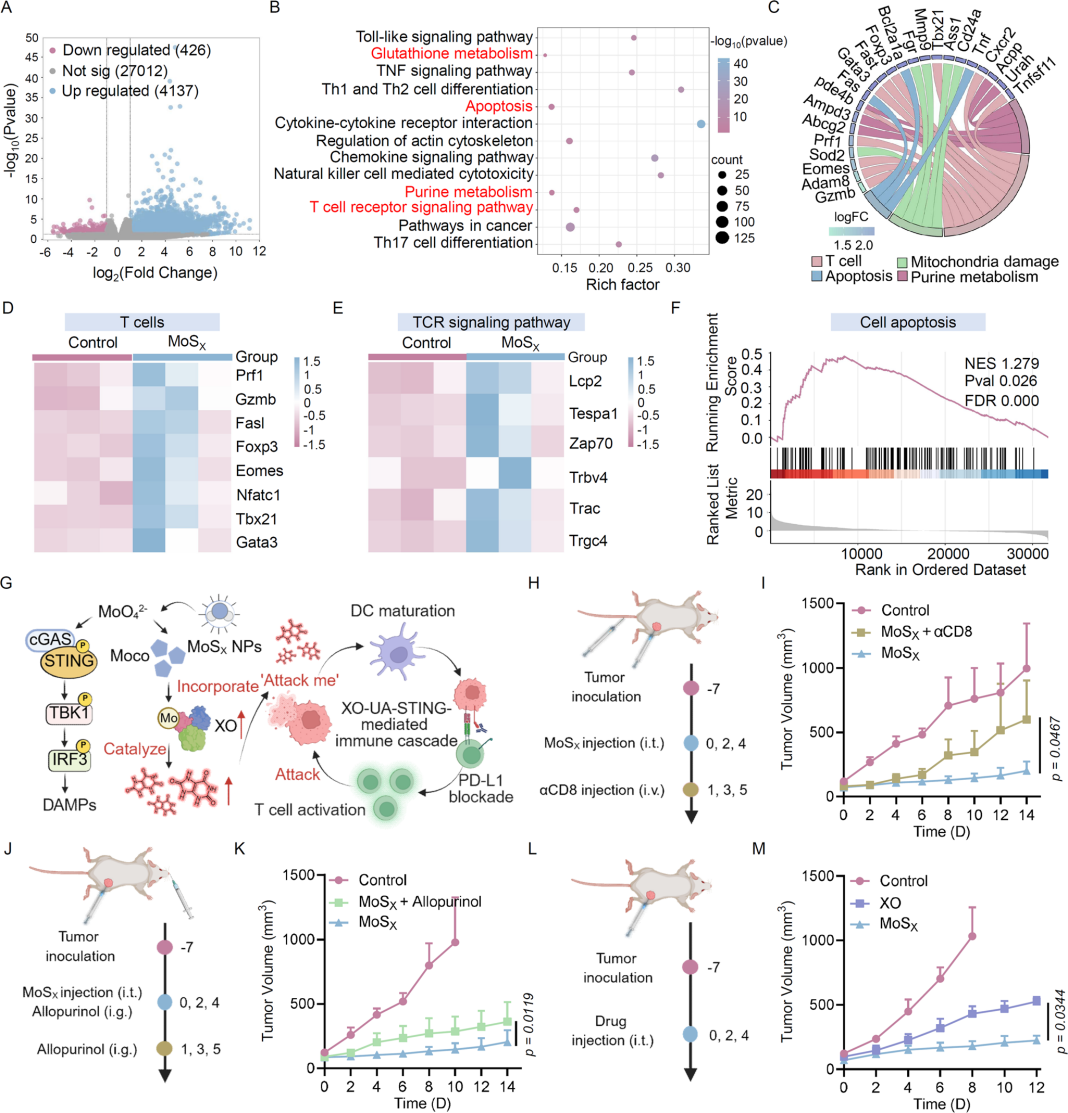
RNA‐Seq analysis of MoS_X_‐treated tumor tissues. (A) Volcano plot of differentially expressed genes before and after MoS_X_ treatment. (B) KEGG pathway enrichment analysis of upregulated differentially expressed genes. (C) Chord plot showing GO term enrichment. (D) Heatmap showing genes up‐regulated during T cell‐mediated immunotherapy. (E) Heatmap of TCR pathway‐related differential genes. (F) GSEA analysis of apoptosis‐related gene sets. (G) Schematic illustration of the underlying therapeutic mechanism of MoS_X_ for DCs maturation, cGAS‐STING pathway activation, improvement in XO activity, and increase in UA content. (H) Schematic diagram of T‐cell blockades for the treatment of CT26 tumor‐bearing mice. (I) Average tumor growth curves of CT26 tumor‐bearing mice after different treatments. (J) Schematic diagram of XO blockade for the treatment of CT26 tumor‐bearing mice. (K) Mean tumor growth curves of CT26 tumor‐bearing mice receiving different treatments. (L) Schematic diagram of XO for the treatment of CT26 tumor‐bearing mice. (M) Average tumor growth curves of CT26 tumor‐bearing mice after different treatments. n.s.: p > 0.05, *p < 0.05, **p < 0.01, ***p < 0.001, and the data are presented as the mean ± SD.

Subsequently, enrichment chord diagrams were constructed to further analyze the functions of these genes (Figure 7C), with a primary focus on purine metabolism, T‐cell related pathways, apoptosis, and mitochondrial dysfunction pathways. The Pde4b gene was shared between the T‐cell pathway and the purine metabolism pathway, and the chord diagram revealed cross‐overlaps, indicating a synergistic effect between these two pathways, which may be accompanied by immune activation and enhanced energy metabolism [47, 48, 49, 50]. Additionally, the significant upregulation of genes associated with T‐cell receptor pathways, which are involved in T‐cell activation, proliferation, and differentiation, usually indicates that T cells are in a state of activation, and this change has important implications for the body's immune response. Specifically, the energy generated by purine metabolism provided the material foundation for T‐cell activation, and conversely, the high activity of T cells further enhanced the demand for purine metabolism.

The upregulation of matrix metalloproteinase 9 (Mmp9) confirmed that MoS_X_ NPs induced mitochondrial dysfunction; in turn, mitochondrial damage further promoted the expression and activation of Mmp9, forming a vicious cycle [51]. Moreover, the downregulation of superoxide dismutase 2 (Sod2), a gene involved in the mitochondrial antioxidant system, also validated the disruption of the mitochondrial membrane structure [52]. Similar to Sod2, glutathione peroxidase 1 (Gpx1), which was involved in regulating mitochondrial function, particularly in managing oxidative stress [53], was also significantly upregulated. This typically represents an adaptive bodily response, which mitigates mitochondrial damage and delays the decline in cellular function by strengthening antioxidant defenses. Furthermore, Gpx1 is linked to the glutathione metabolic cycle; its upregulation leads to substantial consumption of GSH to maintain redox balance. In addition, after MoS_X_ NPs treatment, the expression of Fas (involved in the positive regulation of apoptosis) was upregulated, while the expression of Bcl2ala and other genes involved in the negative regulation of cell proliferation was downregulated. Compared with the control group, the MoS_X_ group exhibited significant differences in the expression of T‐cell related genes and those associated with the TCR signaling pathway (Figure 7D, E). The results of Gene Set Enrichment Analysis (GSEA) for the gene set related to cell apoptosis revealed significant enrichment between the two groups of samples (p < 0.05, FDR < 0.05), indicating that MoS_X_ treatment significantly affected the activity of the cell apoptosis pathway and caused an obvious enrichment trend of genes associated with cell apoptosis (Figure 7F). All of the above results demonstrated that the degradation products of MoS_X_ NPs regulated purine metabolism, activated T‐cell related pathways (MoO_4_^2−^), triggered mitochondrial dysfunction pathways, and then induced cancer cell apoptosis (H_2_S) (Figure 7G).

### 2.9 Verification of the role of MoS_X_ in Specific Metabolic‐Immune Therapy

To further validate the important role of T cells in MoS_X_‐activated antitumor immune responses, an anti‐CD8 antibody (αCD8) was employed to block T‐cell function following MoS_X_ administration. CT26 tumor‐bearing mice were intratumorally injected with MoS_X_ NPs and then intravenously injected with the αCD8 depletion antibody (Figure 7H). Compared with the MoS_X_ monotherapy group, the depletion of CD8^+^ T cells significantly abolished tumor suppression, suggesting that T cells are critical in MoS_X_‐mediated tumor treatment (Figure 7I). Moreover, the T‐cell blocking group still exhibited partial tumor suppression relative to the control group, suggesting that additional anti‐tumor pathways potentially involving cell metabolism or XO catalysis are involved in antitumor process. To further confirm that MoS_X_ is involved in the regulation of purine metabolism, allopurinol was orally administered to inhibit XO catalytic activity and reduce UA synthesis (Figure 7J). The allopurinol also partially abolished the tumor suppressive effect of MoS_X_ (Figure 7K), further indicating that MoS_X_ was involved in purine metabolism and UA production, thereby effectively enhancing the T‐cell mediated anti‐tumor immunity. On the basis of the previous findings, we directly injected XO into tumors to elevate XO levels and evaluated its effect on tumor inhibition. Using a CT26 subcutaneous tumor model, XO was injected into the tumor to replace the MoS_X_ injection (Figure 7L). The tumor growth of the mice in the XO injection group was significantly inhibited, albeit to a lesser extent than that in the MoS_X_ group (Figure 7M), which further confirmed the critical role of XO activity in inhibiting tumor growth and that a continuous supply of XO had better therapeutic effects. Finally, the CD3^+^ and CD8^+^ staining results further revealed increased infiltration of T cells in the XO‐ and MoS_X_‐ injected tumors (Figure S16), indicating a relationship between T cells and XO activity. Collectively, MoS_X_ exerted efficient anti‐tumor effects through increased XO activity and UA production, thereby enhancing T‐cell mediated anti‐tumor immunity.

## 3 Conclusions

In summary, given the important role of molybdenum enzymes and UA, biodegradable MoS_X_ NPs were successfully synthesized via a one‐pot strategy to increase molybdenum enzyme activity and thus effectively potentiate anti‐tumor immunity. This dual‐modality approach not only amplified immune activation but also triggered the STING signaling pathway and modulated purine metabolic networks, thereby orchestrating a comprehensive enhancement of anti‐tumor immune responses. The biodegradable MoS_X_ NPs exhibited excellent GSH‐responsiveness, triggering the rapid release of H_2_S and MoO_4_^2−^. On the one hand, H_2_S induced mitochondrial dysfunction, which promoted the release of mtDNA. This mtDNA then synergizes with MoO_4_^2−^ to activate the cGAS‐STING pathway, thereby driving the maturation of DCs. On the other hand, as the catalytic moiety of molybdenum, MoO_4_^2−^ regulated cellular purine metabolic reprogramming and increased UA level in the tumor, thereby achieving synergistic anti‐tumor immune responses. This bidirectional activation strategy upregulated DCs and T‐cell abundance while promoting pro‐inflammatory cytokine release, which significantly assisted in ICB immunotherapy. Overall, the developed MoS_X_ NPs can reshape the tumor microenvironment via the dual effects of acid‐responsive degradation and GSH depletion. Moreover, this nanosystem is able to reprogramme purine metabolism in tumors and increase UA production to reverse the immunosuppressive microenvironment, thereby effectively triggering robust antitumor immune responses. Notably, MoS_X_ NPs undergo selective degradation in GSH‐abundant tumor tissues, which minimizes off‐target effects and enhances tumor‐specific accumulation and delivery. This study proposes a metal‐based nanocatalytic strategy to improve purine metabolic networks, activate T cells, and trigger a strong anti‐tumor response, thereby achieving precision tumor metabolic‐immune therapy and providing new insights into tumor prevention and treatment.

Although the antitumor efficacy of the MoS_X_ NPs was promising in the present study, several limitations remain to be improved and further explored. On the one hand, we did not directly verify whether molybdenum was successfully incorporated into Moco through experiments. Instead, this conclusion was only indirectly supported by the relevant literature, which restricts the rational optimization of Moco generation efficiency in future research. On the other hand, this study focused only on the upregulation of XO activity among molybdenum enzymes, whereas the activity changes of other molybdenum enzyme family members (such as AO and SO) were not systematically investigated. Consequently, the overall regulatory effect of MoS_X_ NPs on the entire molybdoenzyme system remains unclear, representing a notable limitation of this work. By addressing these limitations, we can further strengthen the mechanistic rationale of this strategy and promote the translational advancement of molybdenum‐based nanomedicines for oncological applications.

## Author Contributions

**Xiaoxiao Pan**: conceptualization, investigation, Writing – original draft. **Yuqi Yang**: conceptualization, investigation. **Zifan Pei**: conceptualization, investigation, writing – original draft. **Nan Jiang**: conceptualization, data curation. **Jie Cao**: conceptualization, investigation. **Zhicheng Liu**: conceptualization, investigation. **Yechen Huang**: conceptualization, investigation. **Qian Li**: data curation, formal analysis. **Qialu Du**: conceptualization, investigation. **Lin Zhang**: software, data curation. **Liang Cheng**: supervision, project administration, writing – review and editing, funding acquisition, investigation. **Fei Gong**: supervision, project administration, writing – review and editing. **Jie Wu**: conceptualization, investigation. **Jinhua Zhou**: software, funding acquisition. **Shumin Sun**: conceptualization, investigation.

## Conflicts of Interest

The authors declare no conflicts of interest.

## Supporting information




**Supporting File**: advs76726‐sup‐0001‐SuppMat.docx.

## Data Availability

The data that support the findings of this study are available from the corresponding author upon reasonable request.
