# Supplementary material for: Engineering‐Modulated Molybdenum Enzymes Strategy for Tumor‐Specific Metabolic‐Immunotherapy
Source: Adv Sci (Weinh). 2026 Jul 23;13(57):e76726. doi: 10.1002/advs.76726 (PMC13393259; doi:10.1002/advs.76726)
Supplement: Supplementary file 1 [file ADVS-13-e76726-s001.docx]

Copyright WILEY-VCH Verlag GmbH & Co. KGaA, 69469 Weinheim, Germany, 2024.

Supporting Information

**Engineering-Modulated Molybdenum Enzymes Strategy for Tumor-Specific Metabolic-Immunotherapy**

*Xiaoxiao Pan^#[a]^, Zifan Pei^#[a]^, Jie Wu^#[a]^, Nan Jiang^#[b]^, Qian Li^[c]^, Yuqi Yang^[a]^, Jie Cao^[a]^, Yechen Huang^[a]^, Shumin Sun^[a]^, Qialu Du^[a]^, Zhicheng Liu^[a]^, Lin Zhang^[b]^, Fei Gong*^[a]^, Jinhua Zhou*^[c]^, and Liang Cheng*^[a]^*

[a]Miss. X. Pan, Dr. Z. Pei, Dr. J. Wu, Dr. Y. Yang, Dr. J. Cao, Miss. Y. Huang, Dr. S. Sun,

Mr. Q. Du, Mr. Z. Liu, Prof. F. Gong, Prof. L. Cheng

Institute of Functional Nano & Soft Materials (FUNSOM),

Biomedical-BasicResearch-Center (BBRC) of Jiangsu Province,

Soochow University, Suzhou, China

E-mails: lcheng2@suda.edu.cn; [gongfei@suda.edu.cn](mailto:gongfei@suda.edu.cn)

[b]Dr. N. Jiang

Department of Interventional Radiology,

The First Affiliated Hospital of Soochow University,

Suzhou 215006, China.

[c]Miss Q Li, Dr. L. Zhang, and Prof. J. Zhou,

Department of Obstetrics and Gynecology,

The First Affiliated Hospital of Soochow University,

Suzhou 215006, China.

E-mail: [fyyzjh@suda.edu.cn](mailto:fyyzjh@suda.edu.cn)

^#^X. Pan, Z. Pei, J. Wu, and N. Jiang contributed equally to this work.

**Experimental Sections**

***Comparison of weak versus strong anti-tumor immunity***

Tumor-bearing mice were divided into two groups based on tumor size: 1) strong anti-tumor immunity and 2) weak anti-tumor immunity. The tumor volumes were recorded every two days. On day 12, the tumors were photographed and weighed. Subsequently, 1 mL of PBS was added to homogenize the samples. T-cell levels were detected by CLSM, while XO activity and UA content were measured using kits; concurrently, IFN-γ levels were determined.

***Materials and reagents***

Molybdenum pentachloride (MoCl_5_, 99.6%), sodium hydrosulfide (NaHS), polyvinylpyrrolidone (PVP, molecular weight 10 kD), and anhydrous ethanol (≥ 99.7%) were purchased from Aladdin, Rhawn, Sigma, and Shanghai Lingfeng Chemical Reagent Co., Ltd., respectively. All chemicals were of analytical grade and were used without further purification.

***Synthesis of MoS_X_ NPs***

MoS_X_ NPs were synthesized via a one-pot method. Briefly, MoCl_5_ (137 mg) was added dropwise to an ethanol-containing mixed aqueous solution of NaHS (56 mg) and PVP (50 mg). The mixture was stirred at room temperature for 4 h, then washed with anhydrous ethanol and centrifuged at 14800 rpm for 5 min. The supernatant was discarded, yielding MoS_X_ NPs.

***Characterizations***

Transmission electron microscopy (TEM) images, elemental mapping, and energy-dispersive X-ray spectroscopy (EDS) spectra of MoS_X_ were obtained using a field emission transmission electron microscope (TALOS 200X). X-ray diffraction (XRD) patterns were recorded using an Empyrean Alpha 1 XRD diffractometer. Raman spectra were acquired using a laser confocal Raman spectrometer (Horiba HR800). The amount of PVP coated on MoS_X_ was determined using a thermogravimetric analyzer (TGA). The hydrodynamic diameter of MoS_X_ was measured using dynamic light scattering (DLS). X-ray photoelectron spectroscopy (XPS) was performed using a Thermo Scientific K-Alpha system. UV-Vis absorption spectra were recorded using a PerkinElmer Lambda 750 UV-NIR spectrophotometer. Molybdenum (Mo) content was determined using inductively coupled plasma optical emission spectrometry (ICP-OES).

***Analysis of H_2_S release***

***Lead acetate dipstick method***
MoS_X_ NPs at different concentrations (0, 0.2, 0.4, 0.8, and 1.6 mM), either alone or in the presence of GSH (1 mM), was added to a 1 mL reaction system in a Petri dish. Lead acetate test strips moistened with water were placed on the Petri dish lid, and the system was sealed and incubated in an oven at 37 °C for 4 h. The color change of the test strips was observed to qualitatively assess H_2_S release.

***WSP-1 probe detection***
MoS_X_ NPs at different concentrations (0, 0.2, 0.4, 0.8, and 1.6 mM) was mixed with GSH (1 mM) and co-incubated with the WSP-1 probe for 2 h. The fluorescence signal of each group was detected using a microplate reader (Ex/Em = 465/515 nm), and fluorescence images were captured using a PerkinElmer small animal *in vivo* fluorescence imaging system to quantitatively analyze H_2_S release.

***Zinc acetate-methylene blue method***
To quantitatively determine H_2_S production, aqueous solutions of MoS_X_ NPs (at different concentrations) and GSH were mixed to form a 1 mL reaction system, which was incubated at 37 °C for 4 h. Then, 1 mL of zinc acetate/sodium acetate solution was added, followed by the addition of DMPD·2HCl and FeCl_3_ to form blue methylene blue (MB). The absorbance at 664 nm was measured, and the H_2_S concentration was calculated using a standard curve generated with NaHS.

***MoO_4_^2-^ ion*** ***release assay***

MoS_X_ alone or in mixtures with GSH was dispersed in PBS and placed in dialysis tubing. After different time points, 20 μL of the liquid sample was pipetted, and the molybdenum content was detected via ICP-MS.

***Cytotoxicity assays***

To determine cell viability, cells (CT26, 4T1, DC2.4, and HUVECs) at a density of 2×10^4^ were seeded in 96-well plates and incubated with different concentrations of NaHS, MoO_4_^2-^, and MoS_X_ for 12 h or other durations. After the addition of MTT and incubation for 4 h, the formazan crystals were solubilized with DMSO, and the absorbance was measured at 490 nm.

***Intracellular H_2_S detection***

CT26 cells at a density of 2×10^5^ were seeded in 12-well plates and incubated for 12 h to ensure cell adhesion. The cells were first treated with NEM (a thiol scavenger) for 15 min, then incubated with NaHS (1 mM), MoO_4_^2-^ (0.5 mM), or MoS_X_ (0.5 mM) for another 8 h. Then, these cells were stained with the WSP-1 probe at 37 °C for 30 min and visualized using confocal laser scanning microscopy (CLSM).

***Mitochondrial function assays***

CT26 cells (2×10^5^) were seeded in 12-well plates and incubated for 12 h. The cells were then treated with MoS_X_ (0.5 mM), GSH (1 mM), or CCCP (10 μL) for 4 h. After removing the medium and washing the cells twice with PBS, the cells were stained with the mitochondrial probe JC-1 for 30 min. Mitochondrial function was assessed via CLSM and flow cytometry.

***Intracellular ROS and GSH detection***

CT26 cells treated with different materials were stained with DCFH-DA (20 μM) or the GSH probe Thiol Tracker Violet (20 μM) for 30 min, and cellular fluorescence was observed using CLSM.

***Apoptosis assays***

CT26 cells (1×10^5^) were seeded in 24-well plates and incubated for 12 h. Then, these cells were incubated with NaHS (1 mM), MoO_4_^2-^ (0.5 mM), or MoS_X_ (0.5 mM) for another 12 h. Next, these cells were stained with an Annexin V-FITC/PI apoptosis detection kit for 30 min, and the apoptosis rates in each group were detected via flow cytometry.

***DC maturation***

Hind leg bones were extracted from 6-week-old C57 mice and cultured in induction medium (containing RPMI-1640, 0.01% GM-CSF, and 0.1% β-mercaptoethanol) for 7 days. To identify bone marrow-derived dendritic cells (BMDCs), cells (1×10^6^ per well) were seeded in untreated 24-well plates and incubated with LPS (1 μg/mL), NaHS, MoO_4_^2-^, or MoS_X_ for 12 h.

***Detection of cGAS-STING pathway-related proteins***

HeLa cells (1×10^7^) were treated with NaHS (1 mM), MoO_4_^2-^ (0.5 mM), or MoS_X_ (0.5 mM) for 12 h. The supernatant was removed, and 0.5 mL of WB and IP cell lysis buffer was added; cells were lysed on ice for 30 min, followed by centrifugation at 14800 rpm for 10 min, after which the supernatant was retained. The protein concentration was determined via BCA quantification. Changes in the expression of cGAS-STING pathway-related proteins in each group were detected via WB.

***Measurement of intracellular UA content***

CT26 cells (2×10^5^) were seeded in 12-well plates and incubated overnight. The cells were then incubated with NaHS (1 mM), MoO_4_^2-^ (0.5 mM), or MoS_X_ (0.5 mM) for 12 h. In the protein-free filtrate, UA reduces phosphotungstic acid under alkaline conditions, yielding tungsten blue, allantoin, and carbon dioxide, and the color intensity is directly proportional to the UA concentration. The intracellular UA content was measured using a commercial kit according to the manufacturer’s protocol.

***Detection of intracellular XO activity***

CT26 cells at a density of 2×10^5^ were seeded in 12-well plates and incubated for 12 h. The cells were then incubated with NaHS (1 mM), MoO_4_^2-^ (0.5 mM), or MoS_X_ (0.5 mM) for 12 h. Xanthine oxidoreductase activity was measured using a commercial kit according to the manufacturer’s protocol.

***T-cell activation assays***

Spleens were extracted from 6-week-old C57 mice. Cells (3×10^4^ per well) were seeded in untreated 24-well plates and incubated with NaHS (1 mM), MoO_4_^2-^ (0.5 mM), or MoS_X_ (0.5 mM) for 12 h. T -cell activation was then identified via flow cytometry.

***Detection of CRT and HMGB1***

CT26 cells at a suitable density were seeded in 12-well plates and incubated for 12 h. The cells were then incubated with NaHS (1 mM), MoO_4_^2-^ (0.5 mM), or MoS_X_ (0.5 mM) for 12 h. To determine HMGB1 release, the cell membranes were permeabilized with 1% Triton solution. The treated cells were subsequently incubated with a CRT primary antibody or a HMGB1 primary antibody for 1 h, followed by incubation at 4 °C for 30 min with an Alexa Fluor 488-conjugated goat anti-rabbit IgG secondary antibody. Finally, the cells were stained with DAPI, and CRT exposure and HMGB1 release were observed using CLSM.

***Detection of ATP content***

CT26 cells at a density of 2×10^5^ were seeded in 12-well plates and incubated for 12 h to ensure cell adhesion. The cells were then incubated with NaHS (0.5 mM), MoO_4_^2-^ (0.5 mM), or MoS_X_ (0.5 mM) for 12 h. The cells were sonicated on ice to disrupt them, followed by centrifugation to collect the supernatant. The ATP content was measured using a commercial kit according to the manufacturer’s protocol.

***MoS_X_ for the treatment of CT26 tumors***

6-week-old female BALB/c mice were subcutaneously injected with 2.5×10^6^ CT26 cells. When the average tumor volume reached 75-100 mm^3^, the mice were randomly divided into four groups (n=5 per group): 1) control, 2) NaHS (intratumoral (i.t.) injection, 40 mM, 50 μL), 3) MoO_4_^2-^ (i.t. injection, 20 mM, 50 μL), and 4) MoS_X_ (i.t. injection, 20 mM, 50 μL). The mice were treated on days 0, 2, and 4. NaHS and MoO_4_^2-^ were treated at the same injection frequency as MoS_X_. The tumor volumes were recorded every 2 days thereafter.

***Photoacoustic imaging of MoS_X_ in tumors***

Three 6-week-old female BALB/c mice were subcutaneously injected with 2.5×10^6^ CT26 cells. When the average tumor volume reached 75-100 mm^3^, the mice were treated with MoS_X_ (i.t. injection, 20 mM, 50 μL), and the retention of MoS_X_ was observed at various time points using a photoacoustic imaging system.

***Molybdenum ion content in tumors***

Female BALB/c mice were subcutaneously injected with 2.5×10^6^ CT26 cells. When the average tumor volume reached 75-100 mm^3^, the mice were randomly divided into five groups based on time points: 12 h, 1 day, 3 days, 5 days, and 7 days, with 3 mice per group. The mice were treated with MoS_X_ (i.t. injection, 20 mM, 50 μL), and tumor tissues were harvested at different time points followed by aqua regia digestion. The molybdenum ion content in the tumor samples was determined via ICP-OES.

***Determination of XO activity and UA content in tumors***

When the mean tumor volume reached 75-100 mm^3^, the mice were divided into two groups (n=5 per group): the control group and the MoS_X_ group (i.t. injection, 20 mM, 50 μL). Three days after MoS_X_ treatment, tumors were excised, homogenized, and centrifuged; the supernatant was then collected and assayed using respective kits.

***Section staining***

For hematoxylin and eosin (H&E) staining, as well as immunofluorescence staining for Ki67, CRT, and HMGB1, the mice were sacrificed on day 2, and the tumors were collected. H&E-stained sections were examined under a microscope, while sections stained for Ki67, CRT, and HMGB1 were observed via confocal imaging.

***Immune evaluation***

When the tumors grew to 75-100 mm^3^, the mice were randomly divided into four groups (n=5 per group): 1) control, 2) NaHS (i.t. injection, 40 mM, 50 μL), 3) MoO_4_^2-^ (i.t. injection, 20 mM, 50 μL), and 4) MoS_X_ (i.t. injection, 20 mM, 50 μL). Groups 2-4 received drug treatment on day 0. On day 7, the mice were sacrificed, and their tumors and tumor-draining lymph nodes were harvested. Single-cell suspensions were prepared from these tissues, stained, and analyzed via flow cytometry. Concurrently, tumor supernatants and lymph node supernatants were collected for detection of relevant cytokines.

***Combination therapy***

To establish a bilateral tumor model, CT26 cells were subcutaneously injected into the left (primary tumor) and right (distant tumor) flanks of the mice. When the tumors grew to 75-100 mm^3^, the mice were randomly divided into four groups (n=4 per group): 1) control, 2) αPD-L1 (intravenous injection (i.v. injection), 1 mg/kg), 3) MoS_X_ (i.t. injection, 20 mM, 50 μL), and 4) combination group (αPD-L1 + MoS_X_). The third and fourth groups received MoS_X_ treatment on days 0, 2, and 4, while the second and fourth groups were injected with αPD-L1 on days 1, 3, and 5. The tumor volumes were recorded every 2 days thereafter. Sections stained for αPD-L1, CD3^+^, and CD8^+^ were observed via confocal imaging.

***T-cell blockade***

To determine the effect of MoS_X_ on T-cell activation, a subcutaneous CT26 tumor model was established via T-cell blockade. When the tumors grew to 75-100 mm^3^, the mice were randomly divided into three groups (n=4 per group): 1) control, 2) MoS_X_ (i.t. injection, 20 mM, 50 μL), and 3) MoS_X_ + αCD8 (i.v. injection, 1 mg/kg). The second and third groups received MoS_X_ treatment on days 0, 2, and 4, while the third group was additionally injected with αCD8 on days 1, 3, and 5. The tumor volumes were recorded every two days.

***XO blockade***

To further investigate the ability of MoS_X_ to enhance molybdenum enzyme activity, a subcutaneous tumor model of CT26 cells was established via XO blockade using allopurinol. When the tumors grew to 75-100 mm^3^, the mice were randomly divided into three groups (n=4 per group): 1) control, 2) MoS_X_ (i.t. injection, 20 mM, 50 μL), and 3) MoS_X_ + allopurinol. The second and third groups received MoS_X_ treatment on days 0, 2, and 4, with the third group additionally administered allopurinol for 6 days (5 mg/kg per day). The tumor volumes were recorded every two days.

***RNA-seq analysis***

To investigate the MoS_X_-induced changes in gene expression in mice, differentially expressed genes were analyzed via RNA-Seq. When the tumors grew to 75-100 mm^3^, the mice were randomly divided into two groups (n=3 per group): 1) control and 2) MoS_X_ (i.t. injection, 20 mM, 50 μL). The MoS_X_ group received drug treatment on days 0, 2, and 4; tumor volumes were recorded every two days, and transcriptome analyses were performed on day 16.

***Comparison of the treatment effects between XO and MoS_X_***

To further compare the effects of MoS_X_ and XO, a subcutaneous CT26 tumor model was established. When the tumors grew to 75-100 mm^3^, the mice were randomly divided into three groups (n=4 per group): 1) control, 2) MoS_X_ (i.t. injection, 20 mM, 50 μL), and 3) XO (i.t. injection, 0.5 U per mouse, 50 μL). Drug treatments were administered on days 0, 2, and 4, and tumor volumes were recorded every two days. CD3^+^ and CD8^+^ stained sections were observed using confocal imaging.

***Safety evaluation of MoS_X_***

To further evaluate the *in vivo* toxicity of MoS_X_, a subcutaneous CT26 tumor model was established. When tumor volumes reached 75-100 mm³, the mice were randomly divided into two groups (n =5 per group): 1) control, 2) MoS_X_ (i.v. injection, 8 mg/kg). MoS_X_ was administered on days 0, 2, and 4. On day 7, blood samples were collected for hematological analysis and serum biochemical assays (e.g., ALT, AST, BUN), and major organs including the heart, liver, spleen, lung, and kidney were harvested for H&E staining.

**Statistical analysis**

All the statistical analysis was performed by GraphPad Prism 8.0.2. The results were expressed as the mean ± standard deviation (SD). Significant differences between two groups were determined via t-test. One-way ANOVA analysis was employed for the significant differences among multiple comparisons. Ns represented no significant difference, *p < 0.05, **p < 0.01 and ***p < 0.001.

**Supporting Figures**

**Figure S1.** Quantitative analysis of CD3^+^ (A) and CD8^+^ (B) T cells infiltration.

**Figure S2.** **Characterizations of MoS_X_**. (A) PSD histogram of MoS_X_ NPs. (B) HRTEM image of MoS_X_ NPs. (C) Full XPS spectrum of MoS_X_ NPs. (D) IR spectra of MoS_X_ NPs with or without PVP.

**Figure S3.** **Effects of MoS_X_ and GSH concentrations on H_2_S generation.** (A) The linear relationship between NaHS concentration and absorption intensity. (B) Relationship between H_2_S production and MoS_X_ concentration. (C) Relationship between H_2_S production and GSH concentration.

**Figure S4.** Absorption spectra of the probe after incubation with MoS_X_ NPs and GSH at different concentrations.

**Figure S5.** XPS spectrum of the Mo 3d peak after reaction with GSH.

**Figure S6.** Fluorescence quantification of WSP-1 in the presence or absence of GSH with MoS_X_ NPs.

**Figure S7.** H_2_S release from MoS_X_ NPs in different pH solutions.

**Figure S8.** Fluorescence staining of H_2_S in CT26 cells after different treatments.

**Figure S9.** Analysis of the effects of different treatments on flow cytometry quantification of the mitochondrial fluorescent probe JC-1 (A) and confocal imaging quantification of GSH (B) in CT26 cells. The data are presented as mean ± SD (n = 3).

**Figure S10.** Individual growth curves of primary (A) and distant (B) tumors after different treatments (n = 4).

**Figure S11**. Effects of MoS_X_ treatment on serum liver and kidney function-related indices in mice (n=5). (A-C) Bar graphs showing the serum levels of (A) alanine aminotransferase (ALT), (B) aspartate aminotransferase (AST), and (C) blood urea nitrogen (BUN) in mice after treatment with MoS_X_.

**Figure S12**. Effects of MoS_X_ treatment on complete blood count (CBC) parameters in mice (n=5). (A-H) Bar graphs showing the levels of (A) white blood cell (WBC), (B) red blood cell (RBC), (C) hemoglobin (HGB), (D) hematocrit (HCT), (E) mean corpuscular volume (MCV), (F) mean corpuscular hemoglobin (MCH), (G) mean corpuscular hemoglobin concentration (MCHC), and (H) platelet (PLT) in the peripheral blood of mice after treatment with MoS_X_. n.s.: p > 0.05, *p < 0.05, **p < 0.01, ***p < 0.001, and the data are presented as the mean ± SD.

**Figure S13.** H&E staining of major mouse organs after MoS_X_ treatment.

**Figure S14.** Mean tumor growth curve after different treatments for RNA-Seq (n = 4).

**Figure S15**. Heatmap showing upregulated genes related to mitochondrial function (A) and cell apoptosis (B).

**Figure S16**. Confocal images of tumor tissue slices stained with CD3^+^ and CD8^+^ fluorescent antibodies after various treatments.
